# Development of a real-time PCR for detection of *Staphylococcus pseudintermedius* using a novel automated comparison of whole-genome sequences

**DOI:** 10.1371/journal.pone.0183925

**Published:** 2017-08-31

**Authors:** Koen M. Verstappen, Loes Huijbregts, Mirlin Spaninks, Jaap A. Wagenaar, Ad C. Fluit, Birgitta Duim

**Affiliations:** 1 Department of Infectious Diseases and Immunology, Faculty of Veterinary Medicine, Utrecht University, Utrecht, the Netherlands; 2 Wageningen Bioveterinary Research, Lelystad, the Netherlands; 3 Department of Medical Microbiology, University Medical Centre Utrecht, Utrecht, the Netherlands; Universitatsklinikum Munster, GERMANY

## Abstract

*Staphylococcus pseudintermedius* is an opportunistic pathogen in dogs and cats and occasionally causes infections in humans. *S*. *pseudintermedius* is often resistant to multiple classes of antimicrobials. It requires a reliable detection so that it is not misidentified as *S*. *aureus*. Phenotypic and currently-used molecular-based diagnostic assays lack specificity or are labour-intensive using multiplex PCR or nucleic acid sequencing. The aim of this study was to identify a specific target for real-time PCR by comparing whole genome sequences of *S*. *pseudintermedius* and non-pseudintermedius.Genome sequences were downloaded from public repositories and supplemented by isolates that were sequenced in this study. A Perl-script was written that analysed 300-nt fragments from a reference genome sequence of *S*. *pseudintermedius* and checked if this sequence was present in other *S*. *pseudintermedius* genomes (n = 74) and non-pseudintermedius genomes (n = 138). Six sequences specific for *S*. *pseudintermedius* were identified (sequence length between 300–500 nt). One sequence, which was located in the *spsJ* gene, was used to develop primers and a probe. The real-time PCR showed 100% specificity when testing for *S*. *pseudintermedius* isolates (n = 54), and eight other staphylococcal species (n = 43). In conclusion, a novel approach by comparing whole genome sequences identified a sequence that is specific for *S*. *pseudintermedius* and provided a real-time PCR target for rapid and reliable detection of *S*. *pseudintermedius*.

## Introduction

*Staphylococcus pseudintermedius* is an opportunistic coagulase-positive pathogen in dogs and cats, which generally causes topical infections and otitis externa [1]. Infections in humans occur sporadically [2], and often have a zoonotic origin [3–5]. *S*. *pseudintermedius* is a normal inhabitant of the skin and mucosa and healthy dogs and cats can be carriers [6,7]. Its methicillin-resistant variant–MRSP–is often multi-resistant, and sometimes resistant against all common antimicrobials [8,9], which makes clinical infections with this organism difficult to treat. Multi Locus Sequence Typing (MLST) of MRPS showed a highly clonal population structure, with ST71 as the predominant type in Northern Europe, ST68 in Northern America [10,11] and ST45 being dominant in Asia [12], but the population of methicillin-susceptible *S*. *speudintermedius* has not been studied in detail.

*S*. *pseudintermedius* belongs together with two other species, *S*. *intermedius* and *S*. *delphini*, to the SIG group. These species were long considered to be the same species [10] and were traditionally distinguished by the different hosts they were isolated from. When coagulase-positive staphylococci are isolated from dogs, the conventional biochemical identification cannot reliably differentiate between *S*. *intermedius* and *S*. *aureus*, what resulted in *S*. *intermedius* based on the host species. A molecular diagnostic test using PCR-RFLP of the pta gene was not specific for identification of *S*. *pseudintermedius* [13,14]. Approaches for partial sequence analysis of the *tuf* [15] or 16S rRNA genes [16] and *sodA* and *hsp60* genes [17] were not always conclusive. A conventional multiplex PCR has been described for identification of different coagulase-positive staphylococci, targeting 900 bp of the *nuc* gene, but this gene contains insufficient variation for species-specific detection of *S*. *pseudintermedius* by real-time PCR (RT-PCR) [18]. More recently it was shown that *S*. *pseudintermedius* isolates can reliably be distinguished from other members of the SIG group by MALDI-TOF MS [19,20]. This is an accurate technique, and the costs per sample are low, but the high cost of the mass spectrometer itself makes that it is not available for all laboratories. An easy-to-use PCR targeting a single sequence for species-specific detection of *S*. *pseudintermedius* is currently not available, but PCR equipment is available in many research and diagnostic laboratories, both in developed and developing countries.

As whole-genome sequences (WGS) of *S*. *pseudintermedius* are becoming increasingly available, and now the cost of sequencing is dropping can comparative genome analysis identify novel nucleic acid diagnostic targets, for use in the species-specific detection of *S*. *pseudintermedius*. Until now, a suitable nucleic acid target for PCR was usually selected manually and was limited to genes that are known to be conserved. The aim of this study was to apply a bioinformatics approach on WGS comparisons to automate the discovery of suitable nucleic acid sequences for species-specific identification of *S*. *pseudintermedius* by means of RT-PCR.

## Materials and methods

### Whole-genome sequencing

Fifteen genomes of *S*. *pseudintermedius* and 119 genomes of other staphylococci were downloaded from public repositories at NCBI and DNA-nexus (strain IDs and accession numbers are listed in S1 Table). The set of WGS was supplemented by sequencing 59 clinical isolates of *S*. *pseudintermedius*, five of *S*. *intermedius*, and 15 of *S*. *delphini* isolates from dogs that were isolated at the Veterinary Microbiology Diagnostic Centre, Utrecht University, the Netherlands. This resulted in 74 *S*. *pseudintermedius* and 138 non-pseudintermedius genomes from different hosts (Table 1).

**Table 1:** Number of staphylococcal genome sequences used for sliding-frame analysis and strains for PCR validation.

Species	No. of genomes	Host	No. of strains for PCR validation
*S*. *agentis*	1	Ovine	
*S*. *aureus*	94	human (n = 84), ovine (n = 3), porcine (n = 1), bovine (n = 2), food (n = 4)	5
*S*. *capitis*	1	human	
*S*. *carnosus*	1	meat	
*S*. *delphini*	15	equine (n = 10, ovine (n = 1), rodent (n = 1), delphinidae (n = 1), unknown (n = 2)	15
*S*. *epidermidis*	4	human (n = 3), bovine (1)	
*S*. *equorum*	1	food	5
*S*. *haemolyticus*	2	human	
*S*. *hyicus*	1	porcine	3
*S*. *intermedius*	5	ovine (n = 4), canine (n = 1)	5
*S*. *lugdunensis*	2	human	
*S*. *pasteuri*	1	unknown	
*S*. *pseudintermedius*	74	canine (n = 71), feline (n = 3)	54
*S*. *saprophyticus*	1	human	3
*S*. *schleiferi*	5	canine	4
*S*. *warneri*	1	human	
*S*. *xylosus*	3	ursidae (n = 1), unknown (n = 2)	3
**Total**	**212**		**97**

The species of the clinical isolates were confirmed by MALDI-TOF MS (Bruker Daltonik), and the strains were sequenced using Illumina-sequencing performed at the Utrecht Sequencing Facility (Hubrecht Institute, KNAW, the Netherlands). The quality of the WGS data was assessed with the Checkm tool [21]. Sequence reads were assembled *de novo* using the program SPAdes v3.1.1 [22]. The final number of genomes per bacterial species is summarised in Table 1, and a list with genotype and accession numbers is available in S1 Table. The sequences of household genes 16S rRNA, *tuf*, *sodA*, and *nuc* were extracted from the genomes and aligned to confirm the taxonomy of the genome sequences and their species assignment in GenBank. The MLST sequence types were extracted from the genome sequences of *S*. *pseudintermedius*.

### Genome comparisons

For phylogenetic analysis of the species in the SIG group the core genomes were determined of at least one genome of each *Staphylococcus* species from the strain list. Using the program Roary v3.5.6 [23], a gene presence/absence analysis was determined. A gene was considered to be part of the *Staphylococcus* core genome if at least 99% of the genomes contained this gene with at least 50% identity and 50% overlap. For the core genome comparison a SNP-alignment was performed and a Neighbour-Joining (NJ) tree was constructed using FastTree [24]. *Macrococcus caseolyticus* (GenBank accession AP009484.1) was included as root.

### Sliding-frame genome analysis

In order to identify a sequence that is conserved in *S*. *pseudintermedius*, but not present in other staphylococci an iterative script was written in Perl to compare all genome sequences with a novel sliding-frame approach The genome of *S*. *pseudintermedius* isolate 14S02884-1 was chosen as an *a priori* reference genome. The reference genome was converted to frames by taking the first 300 nt of the reference sequence. Then, the frame of 300 nt shifted 100 nt downstream and the sequence in the frame was also recorded (nucleotides 100–400); this new frame had an overlap of 200 nt with the first frame. This process was continued until the end of the genome was reached and the reference genome was available in 300 nt sequences with 200 nt overlap with the neighbouring frame. First, a BLASTn of all *S*. *pseudintermedius* frames against the database with 138 non-pseudintermedius genomes was performed. If a frame was present in non-pseudintermedius genomes it would be unsuitable for a pseudintermedius-specific target for PCR and was excluded from further analysis. Frames were first compared to non-pseudintermedius genomes because it was shown by the genome comparison described above that all staphylococci have a large part of their genome sequences in common and a large part of the frames could be excluded from further analyses, which saved time. The frames that met the pre-set criteria (Identity score<20 *or* Coverage score<20) were analysed with BLASTn to the first non-reference genome of *S*. *pseudintermedius*, and the remaining frames were iteratively compared to the other *S*. *pseudintermedius* genomes (BLASTn filter was set to 100% identity and 100% query coverage for *S*. *pseudintermedius* genomes). In this way, each iteration consisted of less BLASTn searches than the previous, because frames (sequences) that were not suitable as a PCR target were excluded immediately. See the diagram in S1 Fig for a schematic overview of the approach. The analysis included complete genomes and genome sequences in contigs. The script requires BioPerl and was run using Perl v5.18.2. The script is available on github (https://github.com/koenverst/slidingframe/).

### PCR design and validation

Overlapping frames that were conserved in *S*. *pseudintermedius* genomes and absent in non-pseudintermedius genomes were combined. To confirm that these sequences were not present in other organisms of which the sequence was not included in the local sliding-frame analysis a BLAST search was performed in the online NCBI GenBank nr-database. Primers and a probe were developed using Primer3 software (http://primer3.ut.cc) and were ordered at Integrated DNA Technologies (Belgium).

Included for PCR validation were 97 isolates of *S*. *pseudintermedius* and non-pseudintermedius (Table 1 and S2 Table). All isolates were analysed by MALDI-TOF MS (Bruker Daltonik) to confirm their species before PCRs were performed. DNA was extracted for PCR analysis by suspending 2 colonies of a fresh overnight culture on Columbia Agar with sheep blood (Oxoid, the Netherlands) in 500 μL Tris-EDTA pH 8.0 (Sigma Aldrich, the Netherlands) and incubated at 95°C for 10 min followed by centrifugation at 20,000x*g* for 1 min. Five μL supernatant was used for conventional PCR with GoTaq Green G2 master mix (Promega, the Netherlands) and 500 nM of each primer, or for RT-PCR using the LightCycler 480 Probes Master (Roche, the Netherlands) on a LightCycler 480-II system, with an optimal oligonucleotide concentrations of 300 nM of each primer and 75 nM probe. The cycling program was 2 min enzyme activation at 95°C, followed by 45 cycles of 30 s. denaturation at 95°C, 30 sec annealing at 58°C, and 1 min elongation at 72°C. For conventional PCR 10 min elongation at 72°C before cooling to 4°C, was added.

## Results

From the genomes that were downloaded from GenBank (S1 Table) were the 16S rRNA, *tuf*, *sodA*, and *nuc* sequences aligned to check for the species identification. All the species matched with the species names present in the online database (data not shown). Of the clinical Staphylococcal isolates that were sequenced for this study the initial MALDI-TOF MS identification matched with the species identification based on the WGS data (S1 Table).

### Genome comparison

**Fig 1:**
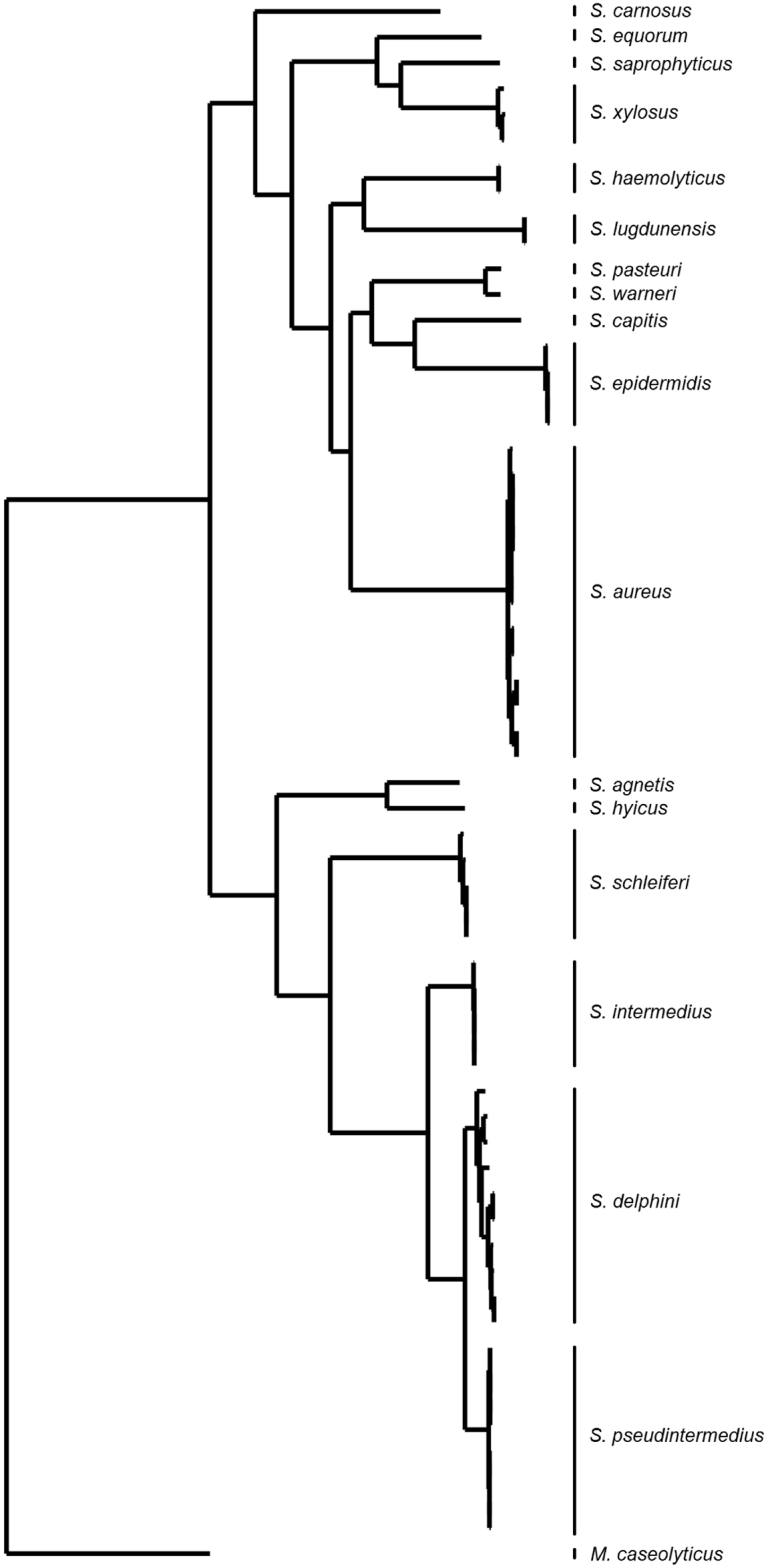
Phylogenetic reconstruction based on comparison of the SNPs in the core-genomes of different staphylococcal species.

A phylogenetic genome comparison of the species of the SIG group and 115 other *Staphylococcus* species confirmed the previously observed genomic relatedness of the SIG species (Fig 1). The staphylococcal core genome consisted of 873 genes. Phylogenetic analysis showed that *S*. *pseudintermedius* is more closely related to *S*. *delphini* than to *S*. *intermedius*. Also, there is only little variation between genomes of *S*. *pseudintermedius*, while *S*. *delphini* genomes are more diverse (Fig 1). The genomes of *S*. *pseudintermedius* belonged to 24 different MLST sequence types (S1 Table).

### PCR target selection by sliding-frame genome analysis

**Fig 2:**
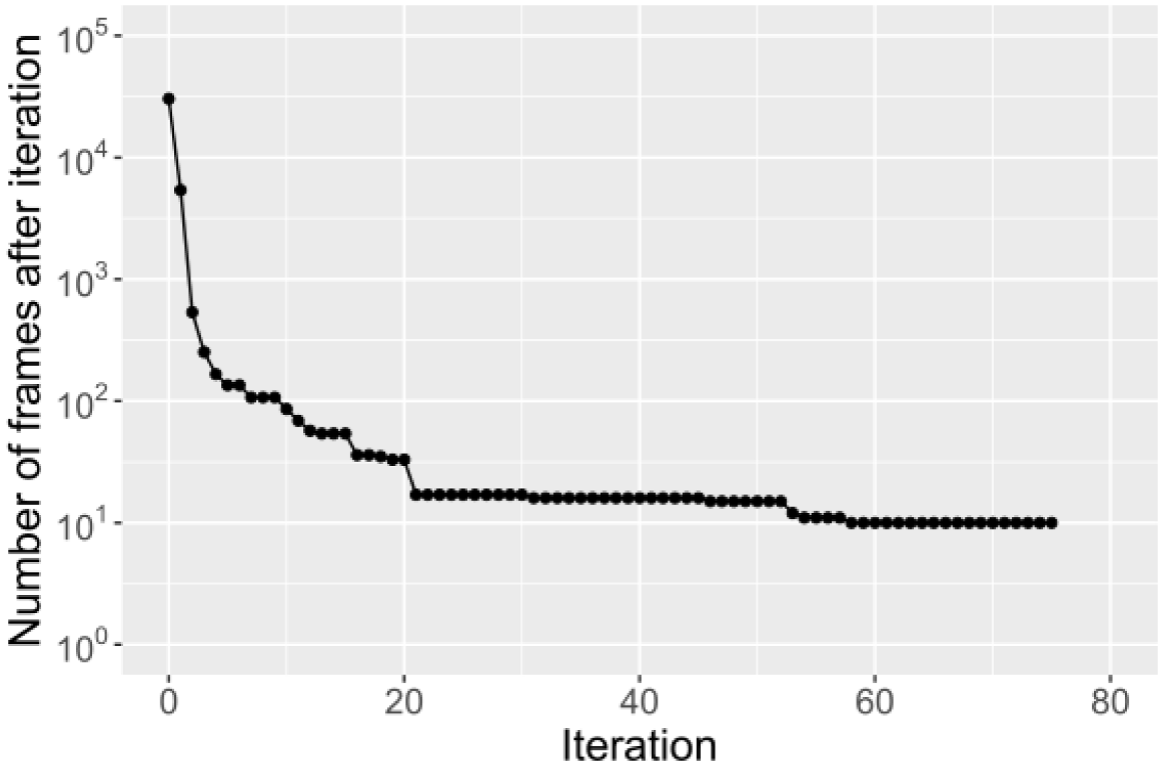
Iteration plot. Number of frames that met the filter criteria after each iteration. At the 0th iteration all frames of the reference genome are present. The 1st iteration is the comparison to the non-pseudintermedius genomes. All following iterations are comparisons to *S*. *pseudintermedius* genomes.

The reference *S*. *pseudintermedius* genome contained a total of 30,391 frames to be analysed when a frame size of 300 and a frame shift of 100 nucleotides were chosen. After the first iteration–comparing the frames (300 nt sequences) of the reference genome to the non-pseudintermedius genomes– 24,992/30,391(80.2%) frames of the *S*. *pseudintermedius* reference genome were found to be present in the genomes of other *Staphylococcus* species (with more than 20% coverage or more than 20% identity) and were not considered in further BLASTn analysis because they would be unsuitable for a *S*. *pseudintermedius*-specific PCR. The remaining 5,399 frames were analysed against the other *S*. *pseudintermedius* genomes to find which frames were conserved in all genome sequences of *S*. *pseudintermedius*. When the script finished, 10 frames were left that met the pre-set filtering criteria. These sequences were identical in all *S*. *pseudintermedius* genomes and not present in genomes of other staphylococci. The number of remaining frames after each iteration is displayed in Fig 2. Overlapping frames could be combined into six sequences ranging from 300–500 nt, and an online BLAST search in the ED99 genome identified the function of the genes in which these sequences were present (Table 2). An online BLAST against all non-pseudintermedius sequences confirmed that the sequences were not present in any other GenBank entry (July 2016).

**Table 2:** *S*. *pseudintermedius*-specific sequences and annotations in strain ED99 that resulted from the sliding-frame analysis.

Seq.	Size (nt)	Annotated proteins in *S*. *pseudintermedius* ED99 (NC_017568.1)
1	400	Teichoic acid translocation permease protein (SPSE_1752)
2	300	Ribonuclease-diphosphate reductase, alpha subunit (*nrdE*) (SPSE_2002)
3	300	Accessory Sec System Translocase (*secY2*) (SPSE_0165) and (*asp1*) (SPSE_0166)
4	500	Hypothetical protein with conserved SDSD and STS motifs (*spsJ*)(SPSE_0164)
5	300	Molybdate ABC transporter, periplasmic molybdate-binding protein (*modA*) (SPSE_0543)
6	400	Multidrug-efflux transporter (SPSE_0785)

The sequences of the six frames were analysed with Primer3 to identify oligonucleotides suitable for conventional and RT-PCR. These oligonucleotides were tested with conventional PCR. This identified that sequence 4 (500 nt) was best suited for PCR amplification. These oligonucleotides target a 198 bp sequence located in position 192532–192684 in the genome of *S*. *pseudintermedius* strain ED99 (GenBank accession NC_017568.1) in the 3’end of the *spsJ* gene. This gene is annotated as a putative cell wall anchor protein in strain ED99, or as hypothetical proteins in other *S*. *speudintermedius* genomes. The following oligonucleotides were used: stapse-Fw: 5'-ACC AAG GCC TGT AAG TAA AGC ACC-3'; stapse-Rev: 5'-TCT CTT TCA ACA TCG GCA TCA ACG C-3', and stapse-P: 5'-6FAM-ACT GTC GCT GAA TCG CTT GAT GAC G-BHQ1-3' as a probe. An online BLAST confirmed that the sequences were specifically detected all *S*. *pseudintermedius* sequences present in GenBank in July 2017.

### PCR validation

The PCR was validated with 54 clinical isolates of *S*. *pseudintermedius* and 43 strains comprising eight staphylococcal species (Table 1). Of these isolates, 16 were reference isolates. The characteristics of the tested isolates are shown in S2 Table. For *S*. *pseudintermedius*, 21 methicillin-susceptible isolates and 33 methicillin-resistant isolates were tested, that were all positive in the RT-PCR. All non-pseudintermedius isolates (n = 43) were negative.

## Discussion

The availability of WGS enabled us to compare genome sequences of *S*. *pseudintermedius* to find a conserved region in the genomes that is not present in other staphylococcal species, for the development of a species-specific PCR.

The core genome comparison showed that *S*. *pseudintermedius* genomes are highly related and are phylogenetically more related to *S*. *delphini* than to *S*. *intermedius*. This correlates with a previous genome comparison based on one isolate per species [10,25,26], and indicates that the close relatedness of strains from the SIG species is sustained after phylogenetic analysis of multiple genome sequences. Genome comparisons with other staphylococcal species confirmed that SIG species belong to a separated phylogenetic branch [27].

Identification of species-specific nucleotides for diagnostic PCR is limited by the availability of publically available genomes of *S*. *pseudintermedius* and related species. Therefore we generated additional genomes in this study, to reduce the risk that the sliding-frame script would identify conserved frames that are not conserved in other *S*. *pseudintermedius* genomes. As *S*. *pseudintermedius* is highly clonal we included the genomes of isolates belonging to 24 different MLST types, that were isolated in different geographical regions (the Netherlands, Switzerland, Japan and Sri Lanka), to cover genomic diversity. With the core genome analysis, no gene was identified that was specific for *S*. *pseudintermedius* and not present in the non-pseudintermedius genomes. The sliding-frame script, identified a six sequences that were species-specific and highly conserved in *S*. *pseudintermedius*, as this approach analysed the nucleotides of the whole genome. From the limited number of nucleic acid sequences that were retrieved from the *S*. *peudintermedius* genomes (i.e. six) only one sequences was suitable for RT-PCR development.

The nucleic acid sequence that was used in this study as PCR target, is located in the *spsJ* gene that encodes one of the 18 putative cell wall-anchored surface proteins in the genome of *S*. *pseudintermedius* strain ED99 [28]. The *spsJ* protein has homology with the *sdrI* gene in *S*. *epidermidis*, and is characterized by conserved motifs SDSD and STS [28]. However, a putative surface location of this protein could not be identified with proteome analysis and further characterization of the function is needed. Therefore is the *spsJ* gene annotated in other *S*. *pseudintermedius* genomes as hypothetical protein. The nucleic acid sequence that is targeted by the PCR is located in the 3’ end of the *spsJ* gene and is conserved and specific for *S*. *pseudintermedius*, and is most likely not under diversifying selection, in contrast to other parts of the encoding region that contain sequence variation (alignment is not shown). A GenBank BLASTn confirmed that the amplified fragment of the *spsJ* gene specifically detects *S*. *pseudintermedius* and is highly conserved in 74 *S*. *pseudintermedius* genomes. This putative diagnostic target was therefore further evaluated for the ability to accurately identify *S*. *pseudintermedius* and to distinguish it from other closely related Staphylococcal species. The RT-PCR was 100% specific for the tested clinical *S*. *pseudintermedius* isolates (n = 54) obtained from dogs (S2 Table). The PCR was not validated for detection of *S*. *pseudintermedius* in clinical samples as diagnostic is not based on a simple-presence of *S*. *speudintermedius*. Dogs can carry *S*. *pseudintermedius* asymptomatically [1], and the in this study developed RT-PCR can quantify the bacteria, enabling to study the association between clinical symptoms and bacterial load, especially when this RT-PCR is applied on samples from potential colonization sites.

Other approaches for genome comparison to identify potential diagnostic targets have been described. In a study on *Haemophilus influenzae* three genome sequences were compared for selection of putative diagnostic PCR targets, and subsequent sequence alignment and development of primers and probes [29]. Although this was a very successful approach, it required a lot of manual work and laboratory resources. The genome comparison approach we described in this study is scalable to large numbers of genome sequences and requires limited manual work once genome sequences are publically available. On the other hand, the approach for comparison of a few genomes, described by Coughlan *et al*. [29], is still very suitable for organisms for which only a few genomes are available in public databases. M-GCAT [30], and MAUVE [31] are also computer programs that are able to compare large numbers of whole genome sequences in a short time, but they can only show sequence similarities and dissimilarities. Our approach automatically filtered sequences that were present in the *S*. *pseudintermedius* genomes and absent in non-pseudintermedius genomes.

In conclusion, we successfully applied a sliding-frame approach for genome comparisons and an automated approach for PCR target selection. Using this approach, we developed a novel RT-PCR assay for species-specific detection of *S*. *pseudintermedius*. This is a promising approach to identify putative diagnostic PCR targets for other organisms.

## Supporting information

S1 Fig Schematic overview of the script. All the frames from the reference genome are analysed with BLAST against the database with non-pseudintermedius staphylococci. The frames that meet the pre-set criteria (i.e. not present in non-pseudintermedius genomes) are iteratively compared to all remaining genomes of *S*. *pseudintermedius*, so that with each iteration less frames need to be analysed.
(PDF)

S1 Table Strain characteristics with accession numbers. (PDF)

S2 Table Clinical isolates and reference strains used for PCR validation. (PDF)
