# Correction: Development of a real-time PCR for detection of *Staphylococcus pseudintermedius* using a novel automated comparison of whole-genome sequences

**DOI:** 10.1371/journal.pone.0189520

**Published:** 2017-12-06

**Authors:** Koen M. Verstappen, Loes Huijbregts, Mirlin Spaninks, Jaap A. Wagenaar, Ad C. Fluit, Birgitta Duim

S1 Table does not contain the complete list of accession numbers. Please see the updated S1 Table below.

## Supporting information

S1 Table Strain characteristics with accession numbers. (PDF)
