# Supplementary material for: Development of a real-time PCR for detection of Staphylococcus pseudintermedius using a novel automated comparison of whole-genome sequences
Source: PLoS One. 2017 Aug 31;12(8):e0183925. doi: 10.1371/journal.pone.0183925 (PMC5578505; doi:10.1371/journal.pone.0183925)
Supplement: S1 Fig — All the frames from the reference genome are analysed with BLAST against the database with non-pseudintermedius staphylococci. The frames that meet the pre-set criteria (i.e. not present in non-pseudintermedius genomes) are iteratively compared to all remaining genomes of S. pseudintermedius, so that with each iteration less frames need to be analysed. (PDF) [file pone.0183925.s001.pdf]

Supplementary data

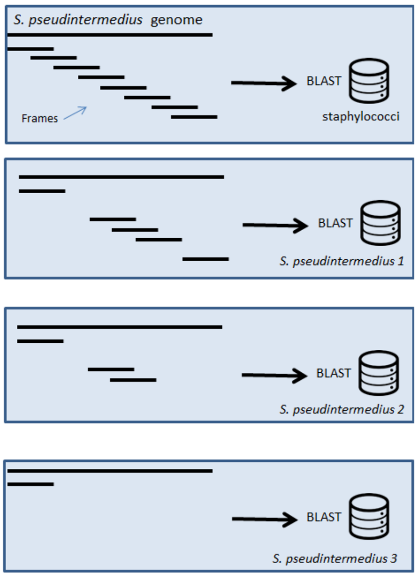

Suppl. 1S Figure: Schematic overview of the script. All the frames from the reference genome are analysed with BLAST against the database with non-pseudintermedius staphylococci. The frames that meet the pre-set criteria (i.e. not present in non-pseudintermedius genomes) are iteratively compared to all remaining genomes of *S. pseudintermedius*, so that with each iteration less frames need to be analysed.
