# Supplementary material for: Development of a real-time PCR for detection of Staphylococcus pseudintermedius using a novel automated comparison of whole-genome sequences
Source: PLoS One. 2017 Aug 31;12(8):e0183925. doi: 10.1371/journal.pone.0183925 (PMC5578505; doi:10.1371/journal.pone.0183925)
Supplement: S1 Table — (PDF) [file pone.0183925.s002.pdf]

Suppl. S1 Table. Strain characteristics of the analyzed genomes

Organism	Strain	lab number	host	MLST Sequence type	Accession	Comment
S. agnetis	908		ovine	n.a	NZ_CP009623.1	
S. aureus	04-02981		human	n.a	NC_017340.1	
S. aureus	08BA02176		human	n.a	NC_018608.1	
S. aureus	2395 USA500		human	n.a	NZ_CP007499.1	
S. aureus	25b MRSA		human	n.a	NZ_CP010299.1	
S. aureus	26b MRSA		human	n.a	NZ_CP010298.1	
S. aureus	27b MRSA		human	n.a	NZ_CP010300.1	
S. aureus	29b MRSA		human	n.a	NZ_CP010295.1	
S. aureus	31b MRSA		human	n.a	NZ_CP010296.1	
S. aureus	33b		human	n.a	NZ_CP010297.1	
S. aureus	502A		human	n.a	NZ_CP007454.1	
S. aureus	Bmb9393		human	n.a	NC_021670.1	
S. aureus	CA12		human	n.a	NZ_CP007672.1	
S. aureus	CA15		human	n.a	NZ_CP007674.1	
S. aureus	CA-347		human	n.a	NC_021554.1	
S. aureus	FCFHV36		human	n.a	NZ_CP011147.1	
S. aureus	FDA209P		human	n.a	NZ_AP014942.1	
S. aureus	HOU1444-VR		human	n.a	NZ_CP012593.1	
S. aureus	HUV05		human	n.a	NZ_CP007676.1	
S. aureus	ILRI_Eymole1/1		porcine	n.a	NZ_LN626917.1	
S. aureus	M121		human	n.a	NZ_CP007670.1	
S. aureus	MI		human	n.a	NZ_AP017320.1	
S. aureus	MS4		human	n.a	NZ_CP009828.1	
S. aureus	MSHR1132		human	n.a	NC_016941.1	
S. aureus	NCTC13435		human	n.a	NZ_LN831036.1	
S. aureus	NCTC8532		human	n.a	NZ_LN831049.1	
S. aureus	NRS 100		human	n.a	NZ_CP007539.1	

S. aureus	RF122		bovine	n.a	NC_007622.1	
S. aureus	RIVM1295		human	n.a	NZ_CP013616.1	
S. aureus	RIVM1607		human	n.a	NZ_CP013619.1	
S. aureus	RIVM3897		human	n.a	NZ_CP013621.1	
S. aureus	RKI4		food	n.a	NZ_CP011528.1	
S. aureus	SA564		human	n.a	NZ_CP010890.1	
S. aureus	HC1335		human	n.a	NZ_CP012012.1	
S. aureus	11819-97		human	n.a	NC_017351.1	
S. aureus	55/2053		human	n.a	NC_022113.1	
S. aureus	6850		human	n.a	NC_022222.1	
S. aureus	ATCC 25923		human	n.a	NZ_CP009361.1	
S. aureus	Be62		human	n.a	NZ_CP012013.1	
S. aureus	CN1		human	n.a	NC_022226.1	
S. aureus	COL		human	n.a	NC_002951.2	
S. aureus	DSM 20231		human	n.a	NZ_CP011526.1	
S. aureus	ECT-R 2		human	n.a	NC_017343.1	
S. aureus	ED133		ovine	133	NC_017337.1	
S. aureus	ED98		ovine	n.a	NC_013450.1	
S. aureus	FORC_001		food	n.a	NZ_CP009554.1	
S. aureus	GR2		human	n.a	NZ_CP010402.1	
S. aureus	Gv51		unknown	n.a	NZ_CP012015.1	
S. aureus	Gv69		unknown	n.a	NZ_CP009681.1	
S. aureus	Gv88		unknown	n.a	NZ_CP012018.1	
S. aureus	HC1340		human	n.a	NZ_CP012011.1	
S. aureus	H-EMRSA-15		human	n.a	NZ_CP007659.1	
S. aureus	HO 5096 0412		human	n.a	NC_017763.1	
S. aureus	JH1		human	n.a	NC_009632.1	
S. aureus	JH9		human	n.a	NC_009487.1	
S. aureus	JKD6008		human	n.a	NC_017341.1	
S. aureus	JKD6159		human	n.a	NC_017338.1	
S. aureus	JS395		human	n.a	NZ_CP012756.1	
S. aureus	LGA251		bovine	n.a	NC_017349.1	

S. aureus	M013		human	n.a	NC_016928.1	
S. aureus	MRSA252		human	n.a	NC_002952.2	
S. aureus	MSSA476		human	n.a	NC_002953.3	
S. aureus	Mu3		human	n.a	NC_009782.1	
S. aureus	Mu50		human	n.a	NC_002758.2	
S. aureus	MW2		human	n.a	NC_003923.1	
S. aureus	N315		human	n.a	NC_002745.2	
S. aureus	NCTC 8325		human	n.a	NC_007795.1	
S. aureus	Newman		human	n.a	NC_009641.1	
S. aureus	SA268		human	n.a	NZ_CP006630.1	
S. aureus	SA40		human	n.a	NC_022443.1	
S. aureus	SA957		human	n.a	NC_022442.1	
S. aureus	ST228 isolate 16035		human	228	NC_020533.1	
S. aureus	ST228 isolate 16125		human	228	NC_020566.1	
S. aureus	ST228 isolate 18583		human	228	NC_020568.1	
S. aureus	ST228 isolate 10388		human	228	NC_020529.1	
S. aureus	ST228 isolate 10497		human	228	NC_020564.1	
S. aureus	ST228 isolate 15532		human	228	NC_020532.1	
S. aureus	ST228 isolate 18341		human	228	NC_020536.1	
S. aureus	ST228 isolate 18412		human	228	NC_020537.1	
S. aureus	ST398		human	398	NC_017333.1	
S. aureus	ST772-MRSA-V DAR4145		human	772	NZ_CP010526.1	
S. aureus	T0131		human	n.a	NC_017347.1	
S. aureus	TCH60		unknown	n.a	NC_017342.1	
S. aureus	TW20		human	239	NC_017331.1	
S. aureus	USA300_2014.C01		human	n.a	NZ_CP012119.1	
S. aureus	USA300_2014.C02		human	n.a	NZ_CP012120.1	
S. aureus	USA300_FPR3757		human	n.a	NC_007793.1	
S. aureus	USA300_TCH1516		human	n.a	NC_010079.1	
S. aureus	VC40		human	n.a	NC_016912.1	
S. aureus	Z172		human	n.a	NC_022604.1	
S. aureus	UA-S391_USA300		human	n.a	NZ_CP007690.1	

S. aureus	USA300-ISMMS1		human	n.a	NZ_CP007176.1	
S. aureus	V2200		human	n.a	NZ_CP007657.1	
S. aureus	XN108		human	n.a	CP007447.1	
S. aureus	XQ		human	n.a	NZ_CP013137.1	
S. capitis	AYP1020		human	n.a	NZ_CP007601.1	
S. carnosus	TM300		meat	n.a	NC_012121.1	
S. delphini	CCUG 38984	14S00091-1	unknown	n.a	MWUN00000000	
S. delphini	IVBBE8	14S02207	unknown	n.a	MWUO00000000	The strain was kindly provided by A. Moodley
S. delphini	AV 8047		ovine	n.a	MWRM00000000	The strain was kindly provided by K. Kikuchi
S. delphini	H4A		equine	n.a	MWRN00000000	The strain was kindly provided by K. Kikuchi
S. delphini	H9-D		equine	n.a	MWRO00000000	The strain was kindly provided by K. Kikuchi
S. delphini	P26		equine	n.a	MWRP00000000	The strain was kindly provided by K. Kikuchi
S. delphini	OD584/10	14S03318-1	rodent	n.a	MWUP00000000	The strain was kindly provided by V. Perreten
S. delphini	KM173/14	14S03319-1	equine	n.a	MWUQ00000000	The strain was kindly provided by V. Perreten
S. delphini	LMG 22190	15S02591-1	delphinida	n.a	MWUR00000000	
S. delphini	214092305301-2		equine	n.a	MWUX00000000	
S. delphini	214092504301-1		equine	n.a	MWUW00000000	
S. delphini	215062304401-1		equine	n.a	MWUV00000000	
S. delphini	215070706401-1		equine	n.a	MWUU00000000	
S. delphini	215100905101-2		equine	n.a	MWUT00000000	
S. delphini	215102607201-2		equine	n.a	MWUS00000000	
S. epidermidis	ATCC 12228		human	n.a	NC_004461.1	
S. epidermidis	PM221		bovine	n.a	NZ_HG813242.1	
S. epidermidis	RP62A		human	n.a	NC_002976.3	
S. epidermidis	SEI		human	n.a	NZ_CP009046.1	
S. equorum	KS1039		food	n.a	NZ_CP013114.1	
S. haemolyticus	JCSC1435		human	n.a	NC_007168.1	
S. haemolyticus	Sh29/312/L2		human	n.a	CP011116.1	
S. hyicus	ATCC 11249		porcine	n.a	NZ_CP008747.1	
S. intermedius	IVBBE5/P66A	14S02204	canine	n.a	MWUY00000000	The strain was kindly provided by V. Perreten
S. intermedius	AV8061		ovine	n.a	MWRQ00000000	The strain was kindly provided by K. Kikuchi

S. intermedius	P4A		ovine	n.a	MWRR00000000	The strain was kindly provided by K. Kikuchi
S. intermedius	P45A		ovine	n.a	MWRS00000000	The strain was kindly provided by K. Kikuchi
S. intermedius	P69A		ovine	n.a	MWRT00000000	The strain was kindly provided by K. Kikuchi
S. lugdunensis	HKU09-01		human	n.a	NC_013893.1	
S. lugdunensis	N920143		human	n.a	NC_017353.1	
S. pasteuri	SP1		unknown	n.a	NC_022737.1	
S. pseudintermedius	E140		canine	71	NZ_ANOI01000001.1	
S. pseudintermedius	ED99		feline	25	NC_017568.1	
S. pseudintermedius	HKU10-03		feline	308	NC_014925.1	
S. pseudintermedius	1726_ED99		feline	260	ERR144844	
S. pseudintermedius	23929		canine	71	ERS140843	
S. pseudintermedius	463949		canine	262	ERS208662	
S. pseudintermedius	69687		canine	309	ERR163420	
S. pseudintermedius	69876		canine	71	ERR144842	
S. pseudintermedius	BNG1		canine	84	ERR144839	
S. pseudintermedius	BNG3		canine	71	ERR144767	
S. pseudintermedius	GL117B		canine	261	ERS217368	
S. pseudintermedius	GL118B		canine	260	ERS217367	
S. pseudintermedius	GL119A		canine	263	ERS208664	
S. pseudintermedius	GL154A		canine	71	ERS208665	
S. pseudintermedius	HH15		canine	71	ERR144844	
S. pseudintermedius	SL/085	14S02692-1	canine	45	MQNB00000000	
S. pseudintermedius	SL/094	14S02708-1	canine	45	MQNC00000000	
S. pseudintermedius	SL/066	14S02752-1	canine	429	MQMO00000000	
S. pseudintermedius	SL/076	14S02760-1	canine	45	MQMP00000000	
S. pseudintermedius	SL/198	14S02793-1	canine	45	MWUZ00000000	
S. pseudintermedius	SL/152	14S02826-1	canine	282	MQNE00000000	
S. pseudintermedius	SL/154	14S02838-1	canine	121	MQNF00000000	
S. pseudintermedius	SL/164	14S02854-1	canine	282	MQNG00000000	
S. pseudintermedius	SL/114	14S02884-1	canine	282	MQND00000000	
S. pseudintermedius	208071704702-1		canine	71	PRJNA393932	
S. pseudintermedius	208071804401-1		canine	71	PRJNA393933	

S. pseudintermedius	208072207201-1		canine	45	PRJNA393934	
S. pseudintermedius	208073001801-1		canine	71	PRJNA393935	
S. pseudintermedius	208081905001-1		canine	71	PRJNA395661	
S. pseudintermedius	208082101701-1		canine	265	PRJNA395662	
S. pseudintermedius	208082803802-1		canine	71	PRJNA395665	
S. pseudintermedius	208090205001-1		canine	261	PRJNA395666	
S. pseudintermedius	208090903101-3		canine	71	PRJNA395667	
S. pseudintermedius	208112602301-1		canine	71	PRJNA395668	
S. pseudintermedius	209011202701-2		canine	71	PRJNA395670	
S. pseudintermedius	209011300901-1		canine	45	PRJNA395671	
S. pseudintermedius	209013002001-1		canine	45	PRJNA395672	
S. pseudintermedius	209020401401-1		canine	71	PRJNA395673	
S. pseudintermedius	209022503501-1		canine	71	PRJNA395674	
S. pseudintermedius	209031201604-5		canine	71	PRJNA395675	
S. pseudintermedius	209032500801-3		canine	71	PRJNA395676	
S. pseudintermedius	209040302601-1		canine	71	PRJNA395677	
S. pseudintermedius	209042702101-1		canine	71	PRJNA395679	
S. pseudintermedius	209052604401-1		canine	71	PRJNA395680	
S. pseudintermedius	209080503802-1		canine	334	PRJNA395681	
S. pseudintermedius	209100702102-1		canine	45	PRJNA395802	
S. pseudintermedius	209113002401-1		canine	261	PRJNA395804	
S. pseudintermedius	210050305001-1		canine	45	PRJNA395805	
S. pseudintermedius	210062301601-1		canine	71	PRJNA395806	
S. pseudintermedius	210081303901-1		canine	258	PRJNA395807	
S. pseudintermedius	210083003701-1		canine	258	PRJNA395808	
S. pseudintermedius	210101902302-1		canine	261	PRJNA395809	
S. pseudintermedius	210102003501-1		canine	336	PRJNA395810	
S. pseudintermedius	211012802302-1		canine	71	PRJNA395811	
S. pseudintermedius	211041505801-1		canine	71	PRJNA395812	
S. pseudintermedius	211083101901-1		canine	71	PRJNA395813	
S. pseudintermedius	212030604001-1		canine	307	PRJNA395814	
S. pseudintermedius	212030802401-1		canine	350	PRJNA395816	

S. pseudintermedius	212042703101-1		canine	71	PRJNA395818	
S. pseudintermedius	212061102701-1		canine	258	PRJNA395819	
S. pseudintermedius	212112902001-1		canine	350	PRJNA395820	
S. pseudintermedius	212122401201-1		canine	258	PRJNA395821	
S. pseudintermedius	213010701401-1		canine	71	PRJNA395822	
S. pseudintermedius	213012202401-1		canine	258	PRJNA395823	
S. pseudintermedius	213012301501-1		canine	342	PRJNA395824	
S. pseudintermedius	213021206001-3		canine	45	PRJNA395825	
S. pseudintermedius	213032704301-1		canine	277	PRJNA395826	
S. pseudintermedius	213041503101-1		canine	346	PRJNA395827	
S. pseudintermedius	213062502301-1		canine	261	PRJNA395828	
S. pseudintermedius	213092504301-1		canine	312	PRJNA395829	
S. pseudintermedius	213101103401-1		canine	258	PRJNA395830	
S. pseudintermedius	213101701201-2		canine	265	PRJNA395832	
S. pseudintermedius	213121103601-1		canine	71	PRJNA395833	
S. pseudintermedius	213123104301-4		canine	45	PRJNA395834	
S. saprophyticus	ATCC 15305		human	n.a	NC_007350.1	
S. schleiferi	1360-13		canine	n.a	NZ_CP009470.1	
S. schleiferi	2142-05		canine	n.a	NZ_CP009762.1	
S. schleiferi	2317-03		canine	n.a	NZ_CP010309.1	
S. schleiferi	5909-02		canine	n.a	NZ_CP009676.1	
S. schleiferi	TSCC54		canine	n.a	NZ_AP014944.1	
S. warneri	SG1		human	n.a	NC_020164.1	
S. xylosus	HKUOPL8		ursidae	n.a	NZ_CP007208.1	
S. xylosus	SMQ-121		unknown	n.a	NZ_CP008724.1	
S. xylosus	C2a		unknown	n.a	NZ_LN554884.1	

n.a. = not available
