# Supplementary material for: Development of a real-time PCR for detection of Staphylococcus pseudintermedius using a novel automated comparison of whole-genome sequences
Source: PLoS One. 2017 Aug 31;12(8):e0183925. doi: 10.1371/journal.pone.0183925 (PMC5578505; doi:10.1371/journal.pone.0183925)
Supplement: S2 Table — (PDF) [file pone.0183925.s003.pdf]

Suppl. S2 Table. Clinical isolates used for PCR validation

Strain number	Species	comment	Host	Source/ Reference	Sample/ Source
213062502301-1	*S. pseudintermedius*	MRSP[1]	Dog	VMDC	Unknown
208071704702-1	*S. pseudintermedius*	MRSP	Dog	VMDC	Unknown
209011202701-2	*S. pseudintermedius*	MRSP	Dog	VMDC	Unknown
V0406964	*S. pseudintermedius*	MRSP	Dog	VMDC	Wound
V0507787	*S. pseudintermedius*	MRSP	Dog	VMDC	Otitis
V0609512	*S. pseudintermedius*	MRSP	Dog	VMDC	Pyoderma
V0613031	*S. pseudintermedius*	MRSP	Dog	VMDC	Pyoderma
V0704501	*S. pseudintermedius*	MRSP	Dog	VMDC	Otitis
V0700233	*S. pseudintermedius*	MRSP	Dog	VMDC	Pyoderma
V0703538	*S. pseudintermedius*	MRSP	Dog	VMDC	Pyoderma
V0708754	*S. pseudintermedius*	MRSP	Dog	VMDC	Wound
V0801939	*S. pseudintermedius*	MRSP	Dog	VMDC	Otitis
V0805157	*S. pseudintermedius*	MRSP	Dog	VMDC	Pyoderma
208080503801-1	*S. pseudintermedius*	MRSP	Dog	VMDC	Wound
208110702701-2	*S. pseudintermedius*	MRSP	Dog	VMDC	Otitis
209122904001-1	*S. pseudintermedius*	MRSP	Dog	VMDC	Pyoderma
209080302201-1	*S. pseudintermedius*	MRSP	Dog	VMDC	Pyoderma
209012301701-1	*S. pseudintermedius*	MRSP	Dog	VMDC	Pyoderma
210052700901-1	*S. pseudintermedius*	MRSP	Dog	VMDC	Wound
210011202701-1	*S. pseudintermedius*	MRSP	Dog	VMDC	Wound
210071301401-1	*S. pseudintermedius*	MRSP	Dog	VMDC	Otitis
210100504301-1	*S. pseudintermedius*	MRSP	Dog	VMDC	Wound
211042603101-1	*S. pseudintermedius*	MRSP	Dog	VMDC	Pyoderma
211011900801-2	*S. pseudintermedius*	MRSP	Dog	VMDC	Wound
211033002401-1	*S. pseudintermedius*	MRSP	Dog	VMDC	Pyoderma
211012702201-1	*S. pseudintermedius*	MRSP	Dog	VMDC	Pyoderma
212082107701-1	*S. pseudintermedius*	MRSP	Dog	VMDC	Pyoderma
212111203001-1	*S. pseudintermedius*	MRSP	Dog	VMDC	Wound
212020103201-1	*S. pseudintermedius*	MRSP	Dog	VMDC	Wound

212020901601-1	*S. pseudintermedius*	MRSP	Dog	VMDC	Pyoderma
213112702201-1	*S. pseudintermedius*	MRSP	Dog	VMDC	Pyoderma
213020703101-1	*S. pseudintermedius*	MRSP	Dog	VMDC	Pyoderma
213021103101-1	*S. pseudintermedius*	MRSP	Dog	VMDC	Pyoderma
208103101301-1	*S. pseudintermedius*	MSSP	Dog	VMDC	Cerumen
208061601601-1	*S. pseudintermedius*	MSSP	Dog	VMDC	Cerumen
209070904201-1	*S. pseudintermedius*	MSSP	Dog	VMDC	Cerumen
209060203801-1	*S. pseudintermedius*	MSSP	Dog	VMDC	Cerumen
209091505401-1	*S. pseudintermedius*	MSSP	Dog	VMDC	Cerumen
210042201601-1	*S. pseudintermedius*	MSSP	Dog	VMDC	Cerumen
210091404601-1	*S. pseudintermedius*	MSSP	Dog	VMDC	Urine
210072603201-2	*S. pseudintermedius*	MSSP	Dog	VMDC	Nares
210033003101-1	*S. pseudintermedius*	MSSP	Dog	VMDC	Skin
211042902102-1	*S. pseudintermedius*	MSSP	Dog	VMDC	Cerumen
211031801801-1	*S. pseudintermedius*	MSSP	Dog	VMDC	Skin
211021504502-1	*S. pseudintermedius*	MSSP	Dog	VMDC	Skin
211041101301-1	*S. pseudintermedius*	MSSP	Dog	VMDC	Cerumen
212110703302-1	*S. pseudintermedius*	MSSP	Dog	VMDC	Skin
212073001901-1	*S. pseudintermedius*	MSSP	Dog	VMDC	Cerumen
212110102201-1	*S. pseudintermedius*	MSSP	Dog	VMDC	Pus
212080904001-1	*S. pseudintermedius*	MSSP	Dog	VMDC	Mouth
213022203002-2	*S. pseudintermedius*	MSSP	Dog	VMDC	Cerumen
213042402601-1	*S. pseudintermedius*	MSSP	Dog	VMDC	Cerumen
213011603101-1	*S. pseudintermedius*	MSSP	Dog	VMDC	Skin
213090502601-1	*S. pseudintermedius*	MSSP	Dog	VMDC	Skin
S61H7	*S. delphini*		Unknown	A. Moodley[2]	Unknown
AV8047	*S. delphini*		Pigeon	V. Perreten[3]	Nares
H-9D	*S. delphini*		Horse	(2)	Nares
P-26	*S. delphini*		Pigeon	(2)	Nares
KM173/14	*S. delphini*		Horse	(1)	Unknown
LMG22190	*S. delphini*		Dolphin	LMG	Skin
CCUG38984	*S. delphini*		Dolphin	CCUG	Skin
H-4A	*S. delphini*		Horse	(2)	Nares

OD584/10	*S. delphini*		Marten	(1)	Lung
214092305301-2	*S. delphini*		Horse	VMDC	Nares
214092504301-1	*S. delphini*		Horse	VMDC	Nares
215062304401-1	*S. delphini*		Horse	VMDC	Skin
215070706401-1	*S. delphini*		Horse	VMDC	Eye
215100905101-2	*S. delphini*		Horse	VMDC	Unknown
215102607201-2	*S. delphini*		Horse	VMDC	Unknown
AV8061	*S. intermedius*		Pigeon	V. Perreten[3]	Nares
P-45A	*S. intermedius*		Pigeon	(2)	Nares
P-69A	*S. intermedius*		Pigeon	(2)	Nares
P66A	*S. intermedius*		Dog	A. Moodley[2]	Unknown
P-4A	*S. intermedius*		Pigeon	(2)	Nares
ATCC35556	*S. aureus*		Unknown	ATCC	Unknown
ATCC29213	*S. aureus*		Unknown	ATCC	Unknown
14S03431-2	*S. aureus*		Unknown	VMDC	Unknown
14S03722-3	*S. aureus*		Unknown	VMDC	Unknown
15S00600-2	*S. aureus*		Unknown	VMDC	Unknown
14S03506-3	*S. epidermidis*		Unknown	VMDC	Unknown
14S03717-4	*S. epidermidis*		Unknown	VMDC	Unknown
15S00592-5	*S. epidermidis*		Unknown	VMDC	Unknown
15S00601-5	*S. epidermidis*		Unknown	VMDC	Unknown
15S01646-5	*S. epidermidis*		Unknown	VMDC	Unknown
LMG13347	*S. schleiferi*		Unknown	LMG	Unknown
LMG22205	*S. schleiferi*		Unknown	LMG	Unknown
LMG19137	*S. schleiferi*		Unknown	LMG	Unknown
DSM6628	*S. schleiferi*		Unknown	V. Perreten[3]	Unknown
14S03578-4	*S. saprophyticus*		Unknown	VMDC	Unknown
15S00601-4	*S. saprophyticus*		Unknown	VMDC	Unknown
15S01643-2	*S. saprophyticus*		Unknown	VMDC	Unknown
DSM20459	*S. hyicus*		Unknown	DSM	Unknown
14S03498-2	*S. hyicus*		Unknown	VMDC	Unknown
14S03716-2	*S. hyicus*		Unknown	VMDC	Unknown
14S03718-2	*S. xylosus*		Unknown	VMDC	Unknown

15S00577-2	*S. xylosus*		Unknown	VMDC	Unknown
15S00924-2	*S. xylosus*		Unknown	VMDC	Unknown

[1] MSSP; methicillin-susceptible *S. pseudintermedius,* MRSP; Methicillin-resistant *S. pseudintermedius*

[2] This strain was kindly provided by A. Moodley, Department of Veterinary Disease Biology, Univeristy of Copenhagen, Denmark.

[3] This strain was kindly provided by V. Perreten, Institute of Veterinary Bacteriology, University of Bern, Switzerlanc

References for bacterial isolates

1. Perreten V, Kadlec K, Schwarz S, Grönlund Andersson U, Finn M, Greko C, Moodley A, Kania SA, Frank LA, Bemis, DA, Franco, A, Lurescia M, Battisti A, Duim B, Wagenaar JA, van Duijkeren E, Weese JS, Fitzgerald JR, Rossano A, Guardabassi L, 2010. Clonal spread of methicillan-resistant *Staphylococcus pseudintermedius* in Europe and Nort America: an international multicentre study. J. Antimicrob. Chemo. 65:1145-1154.

2. Sasaki T, Kikuchi K, Tanaka Y, Takahashi N, Kamata S, Hiramatsu K. 2007. Reclassification of phenotypically identified S*taphylococcus intermedius* strains. J Clin Microbbiol 45:2770 -2778.
