# Supplementary material for: Correction: Development of a real-time PCR for detection of Staphylococcus pseudintermedius using a novel automated comparison of whole-genome sequences
Source: PLoS One. 2017 Dec 6;12(12):e0189520. doi: 10.1371/journal.pone.0189520 (PMC5718433; doi:10.1371/journal.pone.0189520)
Supplement: S1 Table — (PDF) [file pone.0189520.s001.pdf]

Suppl. S1 Table. Strain characteristics of the analyzed genomes

Organism	Strain	lab number	host	MLST Sequence type	Genome Accession number	MALDI-TOF MS score	Species by MALDI-TOF	PCR
S. agnetis	908		ovine	n.a	NZ_CP009623.1	n.a	n.a	-
S. aureus	04-02981		human	n.a	NC_017340.1	n.a	n.a	-
S. aureus	08BA02176		human	n.a	NC_018608.1	n.a	n.a	-
S. aureus	2395 USA500		human	n.a	NZ_CP007499.1	n.a	n.a	-
S. aureus	25b MRSA		human	n.a	NZ_CP010299.1	n.a	n.a	-
S. aureus	26b MRSA		human	n.a	NZ_CP010298.1	n.a	n.a	-
S. aureus	27b MRSA		human	n.a	NZ_CP010300.1	n.a	n.a	-
S. aureus	29b MRSA		human	n.a	NZ_CP010295.1	n.a	n.a	-
S. aureus	31b MRSA		human	n.a	NZ_CP010296.1	n.a	n.a	-
S. aureus	33b		human	n.a	NZ_CP010297.1	n.a	n.a	-
S. aureus	502A		human	n.a	NZ_CP007454.1	n.a	n.a	-
S. aureus	Bmb9393		human	n.a	NC_021670.1	n.a	n.a	-
S. aureus	CA12		human	n.a	NZ_CP007672.1	n.a	n.a	-
S. aureus	CA15		human	n.a	NZ_CP007674.1	n.a	n.a	-
S. aureus	CA-347		human	n.a	NC_021554.1	n.a	n.a	-
S. aureus	FCFHV36		human	n.a	NZ_CP011147.1	n.a	n.a	-
S. aureus	FDA209P		human	n.a	NZ_AP014942.1	n.a	n.a	-
S. aureus	HOU1444-VR		human	n.a	NZ_CP012593.1	n.a	n.a	-
S. aureus	HUV05		human	n.a	NZ_CP007676.1	n.a	n.a	-
S. aureus	ILRI_Eymole1/1		porcine	n.a	NZ_LN626917.1	n.a	n.a	-
S. aureus	M121		human	n.a	NZ_CP007670.1	n.a	n.a	-
S. aureus	MI		human	n.a	NZ_AP017320.1	n.a	n.a	-
S. aureus	MS4		human	n.a	NZ_CP009828.1	n.a	n.a	-
S. aureus	MSHR1132		human	n.a	NC_016941.1	n.a	n.a	-
S. aureus	NCTC13435		human	n.a	NZ_LN831036.1	n.a	n.a	-
S. aureus	NCTC8532		human	n.a	NZ_LN831049.1	n.a	n.a	-

S. aureus	NRS 100		human	n.a	NZ_CP007539.1	n.a	n.a	-
S. aureus	RF122		bovine	n.a	NC_007622.1	n.a	n.a	-
S. aureus	RIVM1295		human	n.a	NZ_CP013616.1	n.a	n.a	-
S. aureus	RIVM1607		human	n.a	NZ_CP013619.1	n.a	n.a	-
S. aureus	RIVM3897		human	n.a	NZ_CP013621.1	n.a	n.a	-
S. aureus	RKI4		food	n.a	NZ_CP011528.1	n.a	n.a	-
S. aureus	SA564		human	n.a	NZ_CP010890.1	n.a	n.a	-
S. aureus	HC1335		human	n.a	NZ_CP012012.1	n.a	n.a	-
S. aureus	11819-97		human	n.a	NC_017351.1	n.a	n.a	-
S. aureus	55/2053		human	n.a	NC_022113.1	n.a	n.a	-
S. aureus	6850		human	n.a	NC_022222.1	n.a	n.a	-
S. aureus	ATCC 25923		human	n.a	NZ_CP009361.1	n.a	n.a	-
S. aureus	Be62		human	n.a	NZ_CP012013.1	n.a	n.a	-
S. aureus	CN1		human	n.a	NC_022226.1	n.a	n.a	-
S. aureus	COL		human	n.a	NC_002951.2	n.a	n.a	-
S. aureus	DSM 20231		human	n.a	NZ_CP011526.1	n.a	n.a	-
S. aureus	ECT-R 2		human	n.a	NC_017343.1	n.a	n.a	-
S. aureus	ED133		ovine	133	NC_017337.1	n.a	n.a	-
S. aureus	ED98		ovine	n.a	NC_013450.1	n.a	n.a	-
S. aureus	FORC_001		food	n.a	NZ_CP009554.1	n.a	n.a	-
S. aureus	GR2		human	n.a	NZ_CP010402.1	n.a	n.a	-
S. aureus	Gv51		unknown	n.a	NZ_CP012015.1	n.a	n.a	-
S. aureus	Gv69		unknown	n.a	NZ_CP009681.1	n.a	n.a	-
S. aureus	Gv88		unknown	n.a	NZ_CP012018.1	n.a	n.a	-
S. aureus	HC1340		human	n.a	NZ_CP012011.1	n.a	n.a	-
S. aureus	H-EMRSA-15		human	n.a	NZ_CP007659.1	n.a	n.a	-
S. aureus	HO 5096 0412		human	n.a	NC_017763.1	n.a	n.a	-
S. aureus	JH1		human	n.a	NC_009632.1	n.a	n.a	-
S. aureus	JH9		human	n.a	NC_009487.1	n.a	n.a	-
S. aureus	JKD6008		human	n.a	NC_017341.1	n.a	n.a	-
S. aureus	JKD6159		human	n.a	NC_017338.1	n.a	n.a	-
S. aureus	JS395		human	n.a	NZ_CP012756.1	n.a	n.a	-

S. aureus	LGA251		bovine	n.a	NC_017349.1	n.a	n.a	-
S. aureus	M013		human	n.a	NC_016928.1	n.a	n.a	-
S. aureus	MRSA252		human	n.a	NC_002952.2	n.a	n.a	-
S. aureus	MSSA476		human	n.a	NC_002953.3	n.a	n.a	-
S. aureus	Mu3		human	n.a	NC_009782.1	n.a	n.a	-
S. aureus	Mu50		human	n.a	NC_002758.2	n.a	n.a	-
S. aureus	MW2		human	n.a	NC_003923.1	n.a	n.a	-
S. aureus	N315		human	n.a	NC_002745.2	n.a	n.a	-
S. aureus	NCTC 8325		human	n.a	NC_007795.1	n.a	n.a	-
S. aureus	Newman		human	n.a	NC_009641.1	n.a	n.a	-
S. aureus	SA268		human	n.a	NZ_CP006630.1	n.a	n.a	-
S. aureus	SA40		human	n.a	NC_022443.1	n.a	n.a	-
S. aureus	SA957		human	n.a	NC_022442.1	n.a	n.a	-
S. aureus	ST228 isolate 16035		human	228	NC_020533.1	n.a	n.a	-
S. aureus	ST228 isolate 16125		human	228	NC_020566.1	n.a	n.a	-
S. aureus	ST228 isolate 18583		human	228	NC_020568.1	n.a	n.a	-
S. aureus	ST228 isolate 10388		human	228	NC_020529.1	n.a	n.a	-
S. aureus	ST228 isolate 10497		human	228	NC_020564.1	n.a	n.a	-
S. aureus	ST228 isolate 15532		human	228	NC_020532.1	n.a	n.a	-
S. aureus	ST228 isolate 18341		human	228	NC_020536.1	n.a	n.a	-
S. aureus	ST228 isolate 18412		human	228	NC_020537.1	n.a	n.a	-
S. aureus	ST398		human	398	NC_017333.1	n.a	n.a	-
S. aureus	ST772-MRSA-V DAR4145		human	772	NZ_CP010526.1	n.a	n.a	-
S. aureus	T0131		human	n.a	NC_017347.1	n.a	n.a	-
S. aureus	TCH60		unknown	n.a	NC_017342.1	n.a	n.a	-
S. aureus	TW20		human	239	NC_017331.1	n.a	n.a	-
S. aureus	USA300_2014.C01		human	n.a	NZ_CP012119.1	n.a	n.a	-
S. aureus	USA300_2014.C02		human	n.a	NZ_CP012120.1	n.a	n.a	-
S. aureus	USA300_FPR3757		human	n.a	NC_007793.1	n.a	n.a	-
S. aureus	USA300_TCH1516		human	n.a	NC_010079.1	n.a	n.a	-
S. aureus	VC40		human	n.a	NC_016912.1	n.a	n.a	-
S. aureus	Z172		human	n.a	NC_022604.1	n.a	n.a	-

S. aureus	UA-S391_USA300		human	n.a	NZ_CP007690.1	n.a	n.a	-
S. aureus	USA300-ISMMS1		human	n.a	NZ_CP007176.1	n.a	n.a	-
S. aureus	V2200		human	n.a	NZ_CP007657.1	n.a	n.a	-
S. aureus	XN108		human	n.a	CP007447.1	n.a	n.a	-
S. aureus	XQ		human	n.a	NZ_CP013137.1	n.a	n.a	-
S. capitis	AYP1020		human	n.a	NZ_CP007601.1	n.a	n.a	-
S. carnosus	TM300		meat	n.a	NC_012121.1	n.a	n.a	-
*S. delphini**	CCUG 38984	14S00091-1	unknown	n.a	MWUN00000000	2.21	*S. delphini*	-
*S. delphini**	IVBBE8	14S02207	unknown	n.a	MWUO00000000	2.16	n.a	-
*S. delphini**	AV 8047		ovine	n.a	MWRM00000000	2.2	n.a	-
*S. delphini**	H4A		equine	n.a	MWRN00000000	2.15	n.a	-
*S. delphini**	H9-D		equine	n.a	MWRO00000000	2.08	*S. delphini*	-
*S. delphini**	P26		equine	n.a	MWRP00000000	2.02	*S. delphini*	-
*S. delphini**	OD584/10	14S03318-1	rodent	n.a	MWUP00000000	2.05	*S. delphini*	-
*S. delphini**	KM173/14	14S03319-1	equine	n.a	MWUQ00000000	2.16	*S. delphini*	-
S. delphini	LMG 22190	15S02591-1	delphinidae	n.a	MWUR00000000	2.09	*S. delphini*	-
S. delphini	214092305301-2		equine	n.a	MWUX00000000	2.1	*S. delphini*	-
S. delphini	214092504301-1		equine	n.a	MWUW00000000	2.09	*S. delphini*	-
S. delphini	215062304401-1		equine	n.a	MWUV00000000	2.7	*S. delphini*	-
S. delphini	215070706401-1		equine	n.a	MWUU00000000	2.15	*S. delphini*	-
S. delphini	215100905101-2		equine	n.a	MWUT00000000	2.11	*S. delphini*	-
S. delphini	215102607201-2		equine	n.a	MWUS00000000	2.16	*S. delphini*	-
S. epidermidis	ATCC 12228		human	n.a	NC_004461.1	n.a	n.a	-
S. epidermidis	PM221		bovine	n.a	NZ_HG813242.1	n.a	n.a	-
S. epidermidis	RP62A		human	n.a	NC_002976.3	n.a	n.a	-
S. epidermidis	SEI		human	n.a	NZ_CP009046.1	n.a	n.a	-
S. equorum	KS1039		food	n.a	NZ_CP013114.1	n.a	n.a	-
S. haemolyticus	JCSC1435		human	n.a	NC_007168.1	n.a	n.a	-
S. haemolyticus	Sh29/312/L2		human	n.a	CP011116.1	n.a	n.a	-
S. hyicus	ATCC 11249		porcine	n.a	NZ_CP008747.1	n.a	n.a	-
S. intermedius~	IVBBE5/P66A	14S02204	canine	n.a	MWUY00000000	n.a	n.a	-

S. intermedius [#]	AV8061		ovine	n.a	MWRQ00000000	n.a	n.a	-
S. intermedius [#]	P4A		ovine	n.a	MWRR00000000	n.a	n.a	-
S. intermedius [#]	P45A		ovine	n.a	MWRS00000000	n.a	n.a	-
S. intermedius [#]	P69A		ovine	n.a	MWRT00000000	n.a	n.a	-
S. lugdunensis	HKU09-01		human	n.a	NC_013893.1	n.a	n.a	-
S. lugdunensis	N920143		human	n.a	NC_017353.1	n.a	n.a	-
S. pasteuri	SP1		unknown	n.a	NC_022737.1	n.a	n.a	-
S. pseudintermedius	E140		canine	71	NZ_ANOI01000001.1	n.a	n.a	+
S. pseudintermedius	ED99		feline	25	NC_017568.1	n.a	n.a	+
S. pseudintermedius	HKU10-03		feline	308	NC_014925.1	n.a	n.a	+
S. pseudintermedius	1726_ED99		feline	260	ERR144844	n.a	n.a	+
S. pseudintermedius	23929		canine	71	ERS140843	n.a	n.a	+
S. pseudintermedius	463949		canine	262	ERS208662	n.a	n.a	+
S. pseudintermedius	69687		canine	309	ERR163420	n.a	n.a	+
S. pseudintermedius	69876		canine	71	ERR144842	n.a	n.a	+
S. pseudintermedius	BNG1		canine	84	ERR144839	n.a	n.a	+
S. pseudintermedius	BNG3		canine	71	ERR144767	n.a	n.a	+
S. pseudintermedius	GL117B		canine	261	ERS217368	n.a	n.a	+
S. pseudintermedius	GL118B		canine	260	ERS217367	n.a	n.a	+
S. pseudintermedius	GL119A		canine	263	ERS208664	n.a	n.a	+
S. pseudintermedius	GL154A		canine	71	ERS208665	n.a	n.a	+
S. pseudintermedius	HH15		canine	71	ERR144844	n.a	n.a	+
S. pseudintermedius	SL/085	14S02692-1	canine	45	MQNB00000000	2.08	*S. pseudintermedius*	+
S. pseudintermedius	SL/094	14S02708-1	canine	45	MQNC00000000	2.1	*S. pseudintermedius*	+
S. pseudintermedius	SL/066	14S02752-1	canine	429	MQMO00000000	2.01	*S. pseudintermedius*	+
S. pseudintermedius	SL/076	14S02760-1	canine	45	MQMP00000000	2.09	*S. pseudintermedius*	+
S. pseudintermedius	SL/198	14S02793-1	canine	45	MWUZ00000000	2.21	*S. pseudintermedius*	+
S. pseudintermedius	SL/152	14S02826-1	canine	282	MQNE00000000	2.23	*S. pseudintermedius*	+
S. pseudintermedius	SL/154	14S02838-1	canine	121	MQNF00000000	2.07	*S. pseudintermedius*	+
S. pseudintermedius	SL/164	14S02854-1	canine	282	MQNG00000000	2.16	*S. pseudintermedius*	+
S. pseudintermedius	SL/114	14S02884-1	canine	282	MQND00000000	2.23	*S. pseudintermedius*	+
S. pseudintermedius	208071704702-1		canine	71	PEOH00000000	2	*S. pseudintermedius*	+

S. pseudintermedius	208071804401-1		canine	71	PEOI00000000	2.11	*S. pseudintermedius*	+
S. pseudintermedius	208072207201-1		canine	45	PEOJ00000000	2.16	*S. pseudintermedius*	+
S. pseudintermedius	208073001801-1		canine	71	PEOK00000000	2.15	*S. pseudintermedius*	+
S. pseudintermedius	208081905001-1		canine	71	PEOL00000000	2.09	*S. pseudintermedius*	+
S. pseudintermedius	208082101701-1		canine	265	PEOM00000000	2.17	*S. pseudintermedius*	+
S. pseudintermedius	208082803802-1		canine	71	PEON00000000	2.21	*S. pseudintermedius*	+
S. pseudintermedius	208090205001-1		canine	261	PEOO00000000	2.08	*S. pseudintermedius*	+
S. pseudintermedius	208090903101-3		canine	71	PEOP00000000	2.02	*S. pseudintermedius*	+
S. pseudintermedius	208112602301-1		canine	71	PEOQ00000000	2.13	*S. pseudintermedius*	+
S. pseudintermedius	209011202701-2		canine	71	PEOR00000000	1.99	*S. pseudintermedius*	+
S. pseudintermedius	209011300901-1		canine	45	PEOS00000000	2.02	*S. pseudintermedius*	+
S. pseudintermedius	209013002001-1		canine	45	PEOT00000000	2.18	*S. pseudintermedius*	+
S. pseudintermedius	209020401401-1		canine	71	PEOU00000000	2.1	*S. pseudintermedius*	+
S. pseudintermedius	209022503501-1		canine	71	PEOV00000000	2.03	*S. pseudintermedius*	+
S. pseudintermedius	209031201604-5		canine	71	PEOW00000000	2.07	*S. pseudintermedius*	+
S. pseudintermedius	209032500801-3		canine	71	PEOX00000000	2.05	*S. pseudintermedius*	+
S. pseudintermedius	209040302601-1		canine	71	PEOY00000000	2.15	*S. pseudintermedius*	+
S. pseudintermedius	209042702101-1		canine	71	PEOZ00000000	2.12	*S. pseudintermedius*	+
S. pseudintermedius	209052604401-1		canine	71	PEPA00000000	2.42	*S. pseudintermedius*	+
S. pseudintermedius	209080503802-1		canine	334	PEPB00000000	2.42	*S. pseudintermedius*	+
S. pseudintermedius	209100702102-1		canine	45	PEPC00000000	2.4	*S. pseudintermedius*	+
S. pseudintermedius	209113002401-1		canine	261	PEPD00000000	2.43	*S. pseudintermedius*	+
S. pseudintermedius	210050305001-1		canine	45	PEPE00000000	2.06	*S. pseudintermedius*	+
S. pseudintermedius	210062301601-1		canine	71	PEPF00000000	2.19	*S. pseudintermedius*	+
S. pseudintermedius	210081303901-1		canine	258	PEPG00000000	2.16	*S. pseudintermedius*	+
S. pseudintermedius	210083003701-1		canine	258	PEPH00000000	2.06	*S. pseudintermedius*	+
S. pseudintermedius	210101902302-1		canine	261	PEPI00000000	2.07	*S. pseudintermedius*	+
S. pseudintermedius	210102003501-1		canine	336	PEPJ00000000	2.11	*S. pseudintermedius*	+
S. pseudintermedius	211012802302-1		canine	71	PEPK00000000	2.05	*S. pseudintermedius*	+
S. pseudintermedius	211041505801-1		canine	71	PEPL00000000	2.03	*S. pseudintermedius*	+
S. pseudintermedius	211083101901-1		canine	71	PEPM00000000	2.03	*S. pseudintermedius*	+
S. pseudintermedius	212030604001-1		canine	307	PEPN00000000	1.99	*S. pseudintermedius*	+

S. pseudintermedius	212030802401-1		canine	350	PEPO00000000	2.4	*S. pseudintermedius*	+
S. pseudintermedius	212042703101-1		canine	71	PEPP00000000	2.08	*S. pseudintermedius*	+
S. pseudintermedius	212061102701-1		canine	258	PEPQ00000000	2.16	*S. pseudintermedius*	+
S. pseudintermedius	212112902001-1		canine	350	PEPR00000000	2.03	*S. pseudintermedius*	+
S. pseudintermedius	212122401201-1		canine	258	PEPS00000000	2.03	*S. pseudintermedius*	+
S. pseudintermedius	213010701401-1		canine	71	PEPT00000000	2.21	*S. pseudintermedius*	+
S. pseudintermedius	213012202401-1		canine	258	PEPU00000000	2.18	*S. pseudintermedius*	+
S. pseudintermedius	213012301501-1		canine	342	PEPV00000000	1.98	*S. pseudintermedius*	+
S. pseudintermedius	213021206001-3		canine	45	PEPW00000000	2.09	*S. pseudintermedius*	+
S. pseudintermedius	213032704301-1		canine	277	PEPX00000000	2.15	*S. pseudintermedius*	+
S. pseudintermedius	213041503101-1		canine	346	PEPY00000000	2.23	*S. pseudintermedius*	+
S. pseudintermedius	213062502301-1		canine	261	PEPZ00000000	2	*S. pseudintermedius*	+
S. pseudintermedius	213092504301-1		canine	312	PEQA00000000	2.06	*S. pseudintermedius*	+
S. pseudintermedius	213101103401-1		canine	258	PEQB00000000	2.12	*S. pseudintermedius*	+
S. pseudintermedius	213101701201-2		canine	265	PEQC00000000	2.21	*S. pseudintermedius*	+
S. pseudintermedius	213121103601-1		canine	71	PEQD00000000	2.09	*S. pseudintermedius*	+
S. pseudintermedius	213123104301-4		canine	45	PEQE00000000	2.11	*S. pseudintermedius*	+
S. saprophyticus	ATCC 15305		human	n.a	NC_007350.1	2.04	*S. saprophyticus*	-
S. schleiferi	1360-13		canine	n.a	NZ_CP009470.1	2.06	*S. schleiferi*	-
S. schleiferi	2142-05		canine	n.a	NZ_CP009762.1	2.07	*S. schleiferi*	-
S. schleiferi	2317-03		canine	n.a	NZ_CP010309.1	2.06	*S. schleiferi*	-
S. schleiferi	5909-02		canine	n.a	NZ_CP009676.1	2.08	*S. schleiferi*	-
S. schleiferi	TSCC54		canine	n.a	NZ_AP014944.1	2.18	*S. schleiferi*	-
S. warneri	SG1		human	n.a	NC_020164.1	2.01	*S. warneri*	-
S. xylosus	HKUOPL8		ursidae	n.a	NZ_CP007208.1	1.99	*S. xylosus*	-
S. xylosus	SMQ-121		unknown	n.a	NZ_CP008724.1	2.03	*S. xylosus*	-
S. xylosus	C2a		unknown	n.a	NZ_LN554884.1	2.03	*S. xylosus*	-

n.a. = not available

* The strain was kindly provided by A. Moodley

~The strain was kindly provided by V. Perreten

[#]The strain was kindly provided by K. Kikuchi

PCR target sequence 4: 198 bp		
Oligo	Sequence 5'- 3'	Tm (°C)
stapse-Fw	ACCAAGGCCTGTAAGTAAAGCACC	63.1
stapse-Rev	TCTCTTTCAACATCGGCATCAACGC	64.3
stapse-P	6FAM-ACTGTCGCTGAATCGCTTGATGACG-BHQ1	66.7

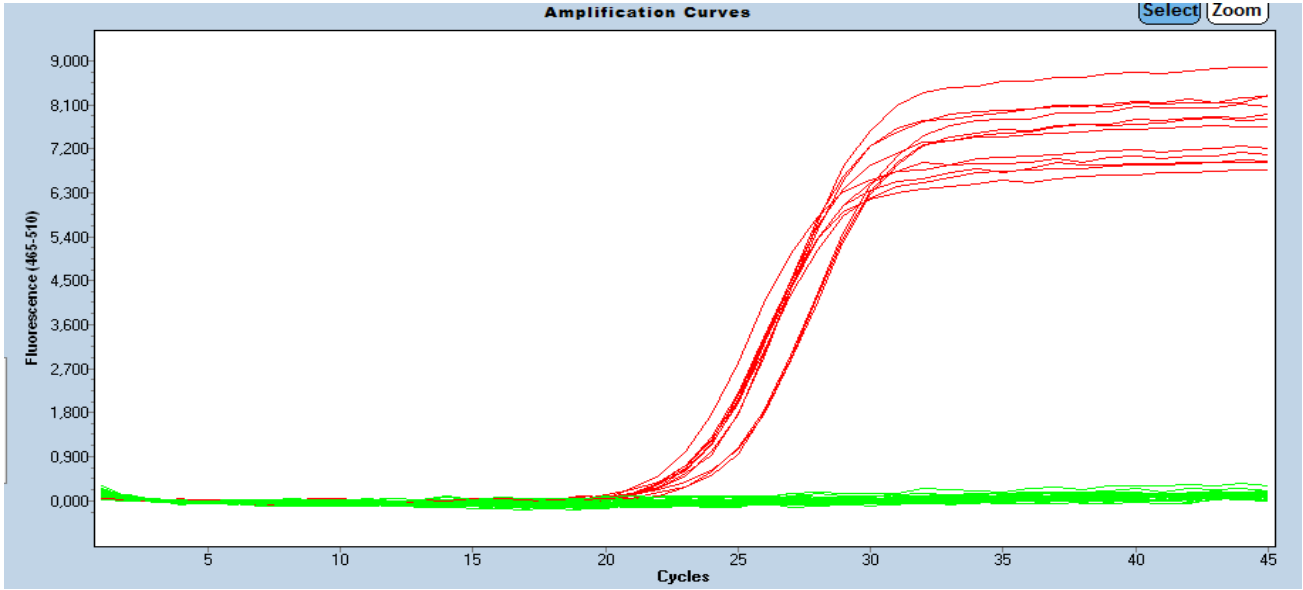

Result for (n=97) strains. Red curves (positive) are all *S. pseudintermedius* strains and green curves (negative) are SIG and other staphylococcal strains.
